# Comparison of the health-related quality of life between epileptic patients with partial and generalized seizure

**Published:** 2014-04-03

**Authors:** Nahid Ashjazadeh, Golnaz Yadollahikhales, Anaheed Ayoobzadehshirazi, Nazanin Sadraii, Negin Hadi

**Affiliations:** 1 Department of Neurology, School of Medicine, Shiraz Neurosciences Research Center, Shiraz University of Medical Sciences, Shiraz, Iran; 2 Department of Community Medicine, School of Medicine, Research Center for Psychiatry and Behavioral Sciences, Shiraz University of Medical Sciences, Shiraz, Iran

**Keywords:** Generalized Epilepsy, Health-Related Quality of Life, Partial Epilepsy

## Abstract

**Background:** Epilepsy is defined as recurrent unprovoked febrile seizures, which cause disability in patients. This study aims to assess the health-related quality-of-life (QOL) in epileptic patients in Fars Province, southern Iran.

**Methods:**One-hundred epileptic patients, above 18 years, referred to Shiraz University of Medical Sciences affiliated clinics, were included. The QOL of patients with generalized and partial seizure were assessed using the Iranian valid and reliable Sf-36 questionnaire. Patients’ socio-demographic and their disease features were also compared with each other using a questionnaire.

**Results:** In partial epilepsy group (n = 24), the married patients in social functioning (SF) aspect of QOL (64.42 ± 14.29) (P = 0.024), the patients on antiepileptic drugs (AEDs) monotherapy in both physical functioning (PF) (88.75 ± 11.57) (P = 0.030) and SF (75.00 ± 6.68) (P = 0.022) aspects, the employed patients in PF aspect of QOL (P = 0.023) (91.87 ± 8.83) and those with high income in mental health aspect of QOL (P = 0.036 and correlation coefficient = 0.413) got better scores compared with the partial epileptic patients who were single, on polytherapy, unemployed and had low to moderate income. In generalized epilepsy group (n = 76), patients on AEDs monotherapy in PF aspect of QOL (P = 0.025) (78.33 ± 24.36) and employed patients in vitality aspect (P = 0.023) (57.00 ± 28.25) had better scores. Data were analyzed using SPSS for windows.

**Conclusion:** Epilepsy can affect patient’s life in a number of ways such as their lives, marriage, occupation, and education. We can encourage patients to find a partner, continue higher education and try to find a job.

## Introduction

Epilepsy is defined as two or more febrile seizures unrelated to acute metabolic disorders or to withdrawal of drugs or alcohol.^1^ It is estimated that the annual incidence of epilepsy is about 50 cases per 100,000 persons (ranging 40–70 per 100,000/year) in developed countries and 100–190 per 100,000/year in developing countries. There is still no definite statistics about the prevalence of the disease among Iranian population.^2^

By involving sensory, motor, autonomic, and psychological systems chronically, epilepsy causes disability in patients.^3^ These problems stem from both the nature of the disease (type of seizure, its frequency, and duration) and the medications used.^4^ The burden of this disease can be overwhelming. Physical injuries such as falls, burns, drowning, and car accidents can threaten the lives of these patients. The fear of having these problems prevents them from going outdoors and doing their house chores.^5^

The patients may need to isolate themselves from the society because people most often stigmatize them.^6^ They limit their visits with family and friends. As a result, they can suffer from depression. These problems can explain why the rate of suicide is high among these patients.^7^ Those mentally affected may lose their self-confidence and feel embarrassed, while facing others. Let aside the disease, the antiepileptic drugs (AEDs) can by itself be a burden on the patients. Their side-effects are fatigue, memory problem, difficulty concentrating, and drowsiness, difficulty in thinking clearly, and nervousness or agitation.^8^

There are several studies examining the quality of life (QOL) of epileptic patients worldwide, especially in developed countries. In a multicenter Italian study, the age and duration of the disease correlated negatively with QOL.^9^ In another study, it was found that the frequency and severity of seizures are the two main determinants in compromising one’s life.^10^ It was also indicated that the type of epilepsy can affect the opinion of people about their QOL. Those with mixed seizures and partial secondary generalized seizure made a lower estimate of their QOL.^8^^,^^11^ However, the information regarding the QOL of epileptic patients in Iran is not satisfactory. Furthermore, due to the impact of multiple factors such as demographic characteristics, socio-economic and clinical presentations, QOL of these patients is different among nations. In spite of the fact that the prevalence of epilepsy is high, few studies have been conducted which have evaluated the QOL in epileptic patients in Iran.^12^^,^^13^ Therefore, we conducted this study in order to assess the QOL in epileptic patients in our region.

## Materials and Methods

The patients were recruited from those referring to Shiraz University of Medical Sciences affiliated clinics. After receiving approval of the medical research Ethics Committee of Shiraz University of Medical Sciences (Approval Number: 89-01-55-2144), written informed consent was obtained from all patients. Sampling was carried out through convenient method. We included patients above 18 years who did not have other comorbidities such as brain degenerative disorders, psychiatric diseases and other mental disabilities (multiple sclerosis, cerebral palsy, brain trauma, stroke, etc.) that would prevent them from answering the questions correctly. The above diseases were excluded by asking the patients if they are a known case of any of them. They were selected from three different levels of income, including low (<2,000,000 Rials monthly), moderate (>2,000,000, <10,000,000 Rials monthly), and high (>10,000,000 Rials monthly). The diagnosis of epilepsy was made by a neurologist based on the patients’ clinical and electroencephalogram data. A total of 100 patients meeting the above criteria were selected and asked to fill out a two-part questionnaire. The first part was about the patients’ demographic and disease features including (age, gender, education, employment status, type and duration of seizure…). The second one was the Iranian valid and reliable Sf-36 questionnaire, asking about their QOL.^14^ Sf-36 measures health-related QOL across eight domains including physical functioning (PF), role functioning, bodily pain (BP), general health (GH), vitality, social functioning (SF), role emotional, and mental health (MH).^15^ The questionnaires were filled out in a self-report manner. The patients were scored from 0 to 100, with 0 having the lowest QOL and 100 the highest. For the purpose of analysis, the patients were divided into two groups: (1) patients with partial seizure and (2) patients with generalized seizures. Data were analyzed using SPSS for windows (version 15; SPSS, Inc., Chicago, IL, USA). The statistical methods used were one-way ANOVA and t-test. Correlations were assessed with a correlation coefficient and Spearman correlation. P value of 0.05 was considered as statistically significant.

## Results

In the recent study, there were 76 patients with generalized epilepsy and 24 with partial epilepsy (39 males and 61 females). Their mean age was 30.33 (± 11.66) and the mean duration of the illness was 3.27, respectively. Majority of patients were also married (57%). The other patient’s demographic characteristics are displayed in table 1.

**Figure 1:**
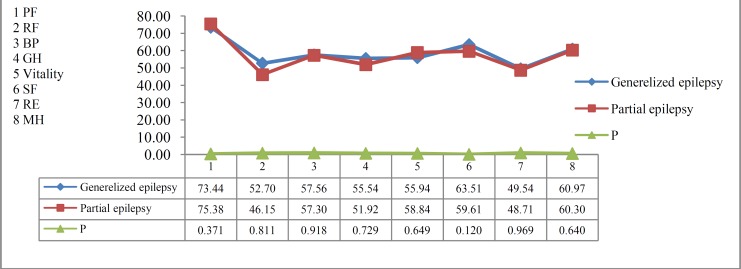
Comparison of quality of life between two groups

**Figure 2:**
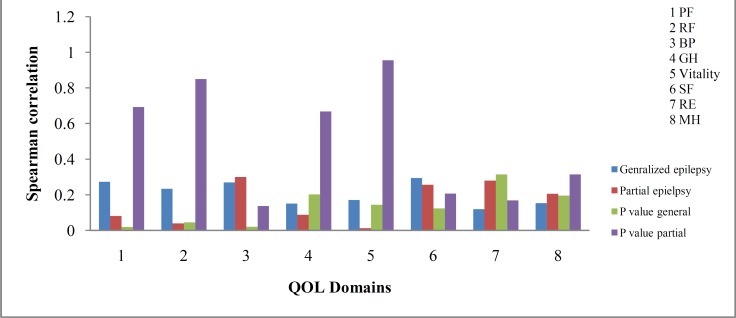
Comparison of quality of life based on education

The results regarding the comparison of the QOL between patients with generalized and partial epilepsy are shown in figure 1. The effect of education, age, gender, and last year attacks on the patient’s QOL is shown in figure 2 and tables 2-4.

Regarding marital status, there is no difference between the QOL in single and married patients in generalized epilepsy group. However, the married patients with partial seizure got better scores in SF aspect of QOL (64.42 ± 14.29) (P = 0.024). Duration of the seizure did not affect the QOL in both groups. Patients with generalized seizure on monotherapy had higher scores in PF aspect of QOL (P = 0.025) (78.33 ± 24.36). This is also true in the partial seizure group in both PF (88.75 ± 11.57) (P = 0.030) and SF (75.00 ± 6.68) (P = 0.022) aspects.

**Table 1:** Socio-demographic characteristics

	**Number**
**Education**	
Illiterate	21
Diploma	53
Bachelor degree	22
Higher than bachelor degree	4
**Income**	
Low	53
Moderate	46
High	1
**Occupation**	
Employed	28
Unemployed	72
**Clinical characteristics**	
**Duration of disease**	
< 1 year	7
1-5 years	17
5–10 years	18
>10 years	58
**Type of medication**	
Monotherapy	50
Polytherapy	50
**Number of attacks in last year**	
No seizure	39
2-3 attack	31
Every month	30

Employed patients with generalized seizure had better QOL in vitality aspect (P = 0.023) (57.00 ± 28.25). On the other hand, employed patients with partial seizure had better scores in PF aspect of QOL (P = 0.023) (91.87 ± 8.83). Those with high income and partial epilepsy showed better scores in MH aspect of QOL (P = 0.036 and correlation coefficient = 0.413).

## Discussion

Epilepsy as a chronic disorder involves the patient in different aspects, i.e., both physically and socially. Their social life becomes compromised due to their loss of self-confidence and the way people treat them. As a result of the repeated seizure attacks, many patients develop motor retardation.^16^ Our results showed that there was no significant difference between the QOL of patients with generalized and partial epilepsy, age affected the QOL of generalized epilepsy group, females and employed patients and those on monotherapy had better QOL in generalized epilepsy group, and duration of seizure had no impact on the QOL of both groups.

**Table 2:** QOL of patients with generalized and partial epilepsy based on gender

**QOL domains**	**Generalized**		**Partial**		**P**		
	**Male**	**Female**	**Male**	**Female**	**Generalized**	**Partial**	
BP	54.23 ± 31.77	59.37 ± 25.04	61.53 ± 29.11	53.07 ± 27.80	0.040		0.701
GH	48.07 ± 29.80	59.58 ± 19.29	54.61 ± 19.73	49.23 ± 31.77	0.001		0.409
Vitality	53.65 ± 27.91	57.18 ± 19.48	59.23 ± 26.28	58.46 ± 21.73	0.020		0.334
PF	73.07 ± 30.98	73.64 ± 27.02	76.92 ± 21.94	73.84 ± 29.16	0.420		0.349
RF	49.03 ± 40.91	54.68 ± 48.41	53.84 ± 46.59	38.46 ± 39.01	0.485		0.215
SF	60.09 ± 33.16	65.36 ± 27.91	56.73 ± 25.31	62.50 ± 23.38	0.422		0.383
RE	38.46 ± 41.83	55.55 ± 39.09	51.28 ± 31.77	46.15 ± 44.17	0.581		0.476
MH	57.07 ± 23.82	63.08 ± 21.47	60.30 ± 20.09	60.30 ± 23.00	0.684		0.612

BP: Bodily pain; GH: General health; PF: Physical functioning; RF: Role functioning; SF: Social functioning; RE: Role emotional; MH: Mental health; QOL: Quality of life

**Table 3:** QOL of patients with generalized and partial epilepsy based on age

**QOL domains**	**Spearman correlation**		**P**	
	**Generalized**	**Partial**	**Generalized**	**Partial**
PF	−0.281	−0.083	0.015	0.686
RF	−0.287	−0.058	0.013	0.780
BP	−0.268	−0.180	0.021	0.379
GH	−0.229	−0.233	0.049	0.251
Vitality	−0.157	−0.170	0.183	0.407
SF	−0.236	0.012	0.043	0.955
RE	−0.381	0.231	0.001	0.257
MH	−0.243	−0.129	0.037	0.530

PF: Physical functioning; RF: Role functioning; BP: Bodily pain; GH: General health; SF: Social functioning; RE: Role emotional; MH: Mental health; QOL: Quality of life

**Table 4:** Comparison of QOL in patients with partial and generalized epilepsy based on last year attacks

**QOL domains**	**No seizure**		**2-3 attacks**		**Every month**		**P**	
	**Generalized**	**Partial**	**Generalized**	**Partial**	**Generalized**	**Partial**	**Generalized**	**Partial**
RF	65.32 ± 39.62	37.50 ± 46.29	51.25 ± 40.12	65.90 ± 40.73	36.95 ± 41.88	25.00 ± 32.27	0.044	0.110
Vitality	62.74 ± 21.32	49.37 ± 18.79	55.50 ± 19.04	71.36 ± 18.17	47.17 ± 25.03	50.00 ± 29.43	0.042	0.063
RE	63.44 ± 40.69	41.66 ± 42.72	41.66 ± 40.28	51.51 ± 37.60	37.68 ± 36.65	52.38 ± 50.39	0.040	0.855
PF	78.54 ± 26.40	60.62 ± 32.99	73.75 ± 22.70	91.36 ± 9.51	66.30 ± 34.15	67.14 ± 20.38	0.292	0.013
BP	64.51 ± 27.54	51.25 ± 30.90	58.00 ± 21.17	72.72 ± 19.54	47.82 ± 30.29	40.00 ± 27.08	0.086	0.036
GH	58.70 ± 23.41	36.87 ± 24.04	58.25 ± 23.41	61.81 ± 22.50	48.91 ± 24.86	53.57 ± 20.14	0.282	0.076
SF	71.37 ± 28.35	56.25 ± 29.88	63.12 ± 23.81	70.45 ± 16.07	53.26 ± 33.96	46.42 ± 22.49	0.085	0.103
MH	65.54 ± 21.43	53.00 ± 20.81	63.60 ± 13.85	67.27 ± 16.47	52,52 ± 27.48	57.71 ± 27.21	0.087	0.338

RF: Role functioning; RE: Role emotional; PF: Physical functioning; BP: Bodily pain; GH: General health; SF: Social functioning; MH: Mental health; QOL: Quality of l

There is a controversy regarding the type of seizure which mostly affects the QOL. Studies by Baker et al.^8^ have indicated that patients suffering from generalized tonic clonic seizures complained of more social problems, both at home and at work. Because they lose their self-esteem, they feel hopeless toward their future. The same result has been found in other studies.^17^^-^^19^ A study among Russian and Indian patients showed that those with localized type seizure had poorer QOL than those with generalized seizure.^16^^,^^20^ Our results indicated that the QOL of patients with generalized epilepsy did not differ statistically from those with partial epilepsy (Figure 1). There is a study by Mohammadi et al.^21^ showing that type of seizure did not correlate significantly with QOL. This result can be attributed to the difference in seizure frequency, duration of the seizure, and its severity in these two groups.

Our findings, similar to many other studies, showed that age correlated well with the QOL of patients with generalized epilepsy (Table 3).^22^^-^^30^ The older patients had the worst QOL. However, there are reports showing that age has no effect on QOL.^11^ Some studies showed that older patients cope better with their epilepsy but have better QOL in some aspects than younger ones. As the patients get older, they face many other chronic problems. Furthermore, they lose their ability to take care of themselves and are not so cooperative in taking their medications or refer to their physicians when needed. The patients with partial epilepsy in this study did not show impaired QOL. This may be due to their having consciousness during seizure attacks and this may decrease the chance of getting hurt. Moreover, many patients with partial epilepsy do not experience severe seizure attacks. Hence, it is possible that they can tolerate it better than those with generalized epilepsy. The difference between the result of our study and that of by Pugh et al.^31^ is due to the fact that we had only one patient aged 66 years and we were not able to evaluate if older patients (>65 years old) have better QOL. Moreover, the variance in the results of our study and others can also stem from the differences between the sample population and the questionnaire used for the evaluation of QOL.^11^

We have also observed that females with generalized epilepsy showed better QOL in three scales (GH, vitality, and BP) compared with males (Table 2). Pugh et al.^31^ and Leidy et al.^32^ have found that females with epilepsy behave better in the physical component of QOL. However, most of the studies showed that men have better QOL compared with women.^4^^,^^19^^,^^33^ Other studies have shown that gender has no relationship with patients’ QOL.^7^^,^^8^ The differences in results can be mainly due to the attitude of different countries toward women and the role women play in their society. It seems that women in Iran pay more attention to their health status and do more routine check-ups than men. Besides, men are more dependent on their wives regarding taking their medication. Furthermore, as men have the main role in making money for their families in Iran, they are more affected by seizure attacks than women who are mostly housewives. As the number of women in our study is twice as much as that of men, it can justify the difference between our results and those of others.^4^^,^^34^

As it has been reported in other studies, those people with generalized epilepsy on monotherapy had better PF and those with partial epilepsy got better scores in both PF and SF.^12^^,^^19^^,^^22^^,^^29^^,^^33^^-^^35^ It is obvious that patients become less cooperative when it comes to using multiple drugs. They may also forget taking their pills. Furthermore, it is also possible that those on polytherapy have more intractable seizure types. So, their poorer QOL can be the result of their more frequent seizure attacks. In addition, polytherapy leads to experiencing more drug adverse effects in patients, which can make them discontinue taking their medication.^12^^,^^29^^,^^35^ It has also been shown that changing one’s medication to monotherapy can improve their QOL tremendously.^33^^,^^36^

The other factor influencing the QOL is employment. The generalized epilepsy group with an occupation had better scores in vitality and those employed patients with partial epilepsy showed better PF. A study by Tlusta et al.^37^ and other scientists^4^^,^^22^^,^^26^^,^^34^ demonstrated the same results. This can result from the feelings of self-confidence and lack of dependency that being employed can give to them. Being unemployed induce feelings of worry about their future and being useless. Moreover, those who are unemployed get bored and face more cognitive problems.^17^ It should not be ignored that epilepsy can greatly affect the patients’ jobs due to the discrimination they feel and the difficulties that may have while working, such as getting hurt by devices at work.^6^^,^^17^ Owing to the fact that educated epileptic patients have a better chance of finding a job, they can also have a better QOL.

We found that the duration of seizure has no correlation with QOL in both groups. Most studies showed that duration of seizure is negatively correlated with QOL.^6^^,^^23^^,^^38^^,^^39^ This can be attributed to their progressive cognitive decline, emotional distress, and the high number of AED.^9^ However, there are some studies, which showed that those experiencing longer duration of seizure have better QOL.^4^^,^^11^ They have postulated that those having longer history of epilepsy coped better with their burden of disease compared with patients who has suffered recently from epilepsy.^4^ This can be due to the difference in the number of patients. Moreover, the duration of seizure in our patients was divided into four groups. So, it is possible that its effect cannot be well-elucidated. However, those who experienced less attacks in the previous year had a better QOL in our study.

Some limitations must be addressed in our study. First, we have gathered our sample from those epileptic patients attending the university affiliated hospital because it was not possible to do randomization. So, they might not be a good representative of the whole society of epileptic patients. Furthermore, the number of patients in our study was not high. The number of epileptic patients with generalized and partial seizure did not equal each other. As a result, their QOL differences might not be shown clearly. As we have excluded the patients with mental and other neurodegenerative diseases from our study, there might be a bias regarding selection of patients. Obviously, the patients who did not have those disabilities had a better QOL.

## Conclusion

We came to the conclusion that epilepsy can affect one’s life in a number of ways. However, our interventions can be most useful in three aspects of their lives, marriage, occupation, and education. First, we can encourage patients to find a partner, continue their higher level education and try to find a job.
